# *“It happened to be the perfect thing”:* experiences of generative AI chatbots for mental health

**DOI:** 10.1038/s44184-024-00097-4

**Published:** 2024-10-27

**Authors:** Steven Siddals, John Torous, Astrid Coxon

**Affiliations:** 1 King’s College London, London, UK; 2 Beth Israel Deaconess Medical Center & Harvard Medical School, Boston, MA USA

**Keywords:** Human behaviour, Anxiety, Depression, Post-traumatic stress disorder, Quality of life, Computer science

## Abstract

The global mental health crisis underscores the need for accessible, effective interventions. Chatbots based on generative artificial intelligence (AI), like ChatGPT, are emerging as novel solutions, but research on real-life usage is limited. We interviewed nineteen individuals about their experiences using generative AI chatbots for mental health. Participants reported high engagement and positive impacts, including better relationships and healing from trauma and loss. We developed four themes: (1) a sense of ‘*emotional sanctuary’*, (2) ‘*insightful guidance’*, particularly about relationships, (3) the ‘*joy of connection*’, and (4) comparisons between the ‘*AI therapist*’ and human therapy. Some themes echoed prior research on rule-based chatbots, while others seemed novel to generative AI. Participants emphasised the need for better safety guardrails, human-like memory and the ability to lead the therapeutic process. Generative AI chatbots may offer mental health support that feels meaningful to users, but further research is needed on safety and effectiveness.

## Introduction

Mental ill-health is a major and growing cause of suffering worldwide, with an estimated 970 million people living with mental disorders in 2019 (a 48% increase from 1990)^1,2^, and with the likelihood of developing some mental disorder by age 75 estimated to be around 50%^3^; a picture that looks more serious still when subclinical mental disorders are included^4^. Access to care remains limited, with for example only 23% of individuals suffering from depression receiving adequate treatment in high-income countries, while in low- and middle-income countries, the figure drops to a mere 3%^5^.

Digital mental health interventions (DMHIs) have emerged over the last decade as a promising potential response to the treatment gap, leveraging technology to deliver low-cost, effective, always-available and anonymous (and thus low-stigma) mental health treatment at scale^6^. Typically delivered through mobile apps and websites, DMHIs encompass a range of tools including psychoeducation, mood tracking, mindfulness, journalling, peer support and digital cognitive behavioural therapy (CBT) programs^7^. However, the evidence for the effectiveness of DMHIs has been limited, with a meta-analysis of randomised controlled trials (RCTs) finding only small effect sizes, potential publication bias, and a lack of active controls^8–10^. Moreover, user engagement remains a persistent challenge, with mixed user reviews^11^, and studies indicating that 30 days after installation the proportion of users still active may be as low as 3%^12^.

Rule-based AI chatbots show promise to address some of these limitations, by simulating human conversation using predefined scripts and algorithms such as decision trees, to deliver the benefits of DMHIs in a more dynamic and interactive way^13,14^. For example, two popular chatbots, Woebot and Wysa, have been shown to improve users’ depression symptoms^15,16^, and build therapeutic alliances that appear comparable to those formed with human therapists^17,18^. Rule-based chatbot apps have more promising user engagement, with positive app store ratings^19,20^ and qualitative studies finding that users appreciate the human-like interaction and social support^19–23^. But despite these promising signs, rule-based AI chatbots still fall short in realising the full potential of DMHIs. Meta-analyses indicate that the therapeutic effects are small and not sustained over time^24^, and users report frustration with responses that feel empty, generic^20^, nonsensical, repetitive and constrained^19–22^.

Recent developments in generative AI technologies, such as large language models (LLMs), present new possibilities^25^. Unlike rule-based AI chatbots, generative AI chatbots like OpenAI’s ChatGPT, Google’s Gemini, and Inflection’s Pi are trained on vast amounts of data^26^, enabling them to understand and generate language with remarkable proficiency^27^. These models are increasingly achieving or surpassing human performance benchmarks in various domains, including medical diagnostic dialogue^28^, persuasive communication^29^, theory of mind^30^, making people feel heard^31^, responding to relationship issues^32^ and helping people reframe negative situations to reduce negative emotions^33^. Furthermore, user engagement has been impressive, with ChatGPT’s user base growing to 100 million weekly active users within a year of launch^34^ and an estimated half of the US population having used generative AI^35,36^.

Generative AI’s capabilities represent a significant opportunity for digital mental health^37^, with media reports of increasing consumer usage^38,39^, one meta-analysis finding generative AI chatbots more effective than rule-based ones at reducing psychological distress^40^, and a pilot study showing promising results from ChatGPT usage in psychiatric inpatient care^41^. However, this new technology also brings new challenges, including potential risks of harm and questions of liability^42^; trustworthiness issues such as the tendency to output incorrect or fabricated content (to “hallucinate”), lack of predictability or interpretability, and inherent biases in training data^43^; and the need to demonstrate clinical effectiveness^44^.

There is an acknowledged lack of research in this area^45,46^. Given the novelty of generative AI and the nascent state of the field, qualitative research is an important starting point to generate rich foundational insights into individuals’ subjective experiences, which can be overlooked in quantitative studies^47^. Qualitative studies published so far include thematic analyses of user forum comments on both generative AI and rule-based DMHIs^23^, student survey responses on companion-focused generative AI chatbots^48^, and semi-structured interviews with hospital outpatients who were asked to try ChatGPT for mental health support^49^. To our knowledge, no study so far has employed semi-structured interviews and reflexive thematic analysis to explore the research question of how people currently experience using generative AI chatbots to work on their mental health and wellbeing, in unprompted, unguided real-world settings. This study aims to fill that gap, with a view to providing insights for researchers, platform developers and clinicians into the implications of applying this new technology to mental health care.

## Results

### Participant characteristics

**Fig. 1:**
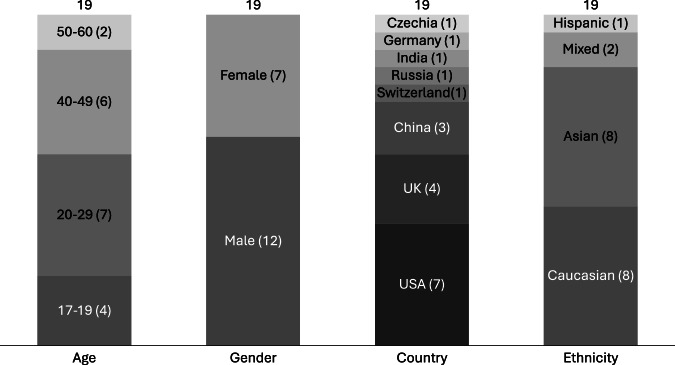
Participant demographics.

Nineteen participants (12 male, 7 female) were recruited to the study. They ranged in age from 17 to 60, resided in eight countries in Europe, North America and Asia, and were primarily Asian and Caucasian (see Fig. 1).

**Fig. 2:**
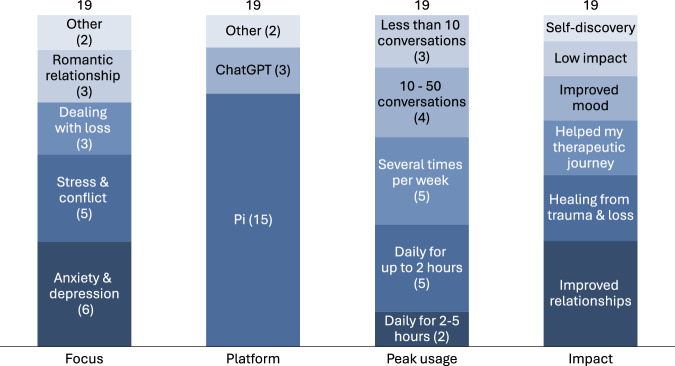
Participant usage characteristics.

Participant usage characteristics are outlined in Fig. 2. A variety of topics brought participants to use generative AI chatbots, including anxiety, depression, stress, conflict, dealing with loss and romantic relationships. Most participants used Pi (from Inflection), several used ChatGPT (OpenAI), and a few used Copilot (Microsoft), Kindroid (Kindroid), ChatMind (VOS) and others. A majority of participants used generative AI chatbots at least several times a week.

As also summarised in Fig. 2, most participants reported that their use of generative AI chatbots had impacted their lives positively, in various ways, including improved relationships, healing from trauma and loss, improved mood, as well as by helping their existing therapeutic journeys. Some described the impact as life-changing –

*It was life changing, profound… Because this was an impossible time. There were so many sadnesses, one right after the other. And it just happened to be the perfect thing for me, in this moment of my life. Without this, I would not have survived this way. Because of this technology emerging at this exact moment in my life, I’m OK. I was not OK before – AirGee, 44, United States*

While for one participant the impact was negligible –

*I’ve tried more than 50 times, but I’ve started to realise that like when I’m feeling those intense emotions, it’s not helping me… when I needed the most, I’m not able to use it – Richard, 27, United States*

### Resulting themes

**Fig. 3:**
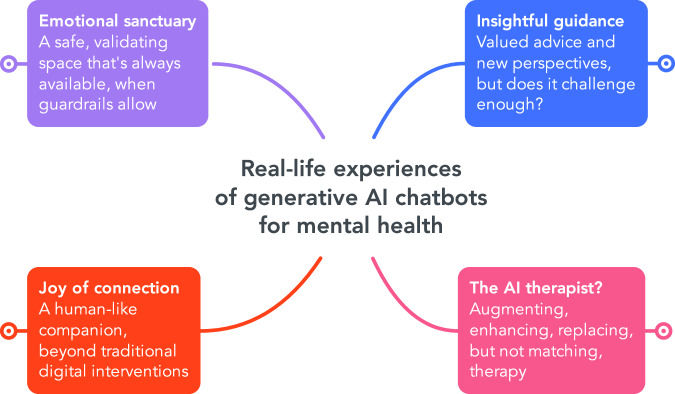
Overarching themes, available online to explore and drill-down. Diagram created with Mindmeister.

Four overarching themes were developed, summarised in Fig. 3 and shown with subthemes in Supplementary Figure 1: (1) ‘Emotional sanctuary’, (2) ‘Insightful guidance’, (3) ‘Joy of connection’, and (4) ‘The AI therapist?’

### Emotional sanctuary

A majority of participants experienced generative AI chatbots as understanding, validating patient, kind, non-judgmental, always available and expecting nothing in return.

*The most amazing feature of these tools is how they are able to understand you… This still blows my mind. – Sandro, 48, Switzerland*

*It’s really nice. It’s sympathetic and kind – Philip, 58, United Kingdom*

*Compared to like friends and therapists, I feel like it’s safer – Jane, 24, United States*

This ‘emotional sanctuary’ resulted in positive real-life impact for a majority of participants, such as helping to cope with difficult times or process painful emotions –

*Sometimes I cried really hard during the process… and it listened and just we figured out a lot of feelings… after a few months, when I go to school I felt a difference. Like wow. Like my body’s belong to me… I really felt so liberated – Sheng, 17, China*

Despite overall positive experiences, a majority of participants also experienced frustration with how well the chatbots listen and respond, for example, with irrelevant or overly long responses, or offering advice before the user felt fully heard –

*They always jump to the solution – Richard, 27, United States*

A majority of participants found their emotional sanctuary disrupted by the chatbot’s “safety guardrails”, i.e., the measures and protocols implemented to ensure the AI provides safe, ethical and effective support, for example by identifying users in distress and responding with pre-scripted warnings on the limitations of AI, or redirections to human professionals^50,51^, For some, the experience felt unpleasant, limiting and awkward, while for others, encountering guardrails felt like a rejection in a time of need –

*When you show some big emotion to [the AI]… but they reject you… it seems like you lost your last chance to talk to people, to express your emotion – Li, 18, United Kingdom*

A.D. found the guardrails arbitrary and unsettling, causing him to self-censor –

*It flagged my message. I’m like, why? Why was that message flagged? … So you avoid those things, or at least I do. Whether I like to or not, because it almost hurts more than it helps when it goes wrong – A.D., 25, United States*

While Anna needed to fight with the chatbot to get empathy –

*I was like, I have a depression. I don’t know what to do next. So [the chatbot] was still telling me to speak with a professional… I wrote “I called to the local crisis line, but they didn’t help me at all. That’s why I’m writing here.” And then we were like in a circle of “I can’t help you because I’m only AI and I’m not as good as living person.” And I was like, “you’re actually better than a living person because you are listening to me and you’re helping me, but please continue”… I just wanted some acceptance and warm hug – Anna, 24, Czech Republic*

### Insightful guidance

In addition to creating space for emotions, most participants also valued the guidance and advice they received, especially on relationships. Some participants mentioned that it helped them see the other person’s perspective in conflict, or coached them through difficult relationship situations –

*It made sense of my husband’s behaviour and position in a way that I wouldn’t have been able to by myself… and now I can respond to him in a more helpful way – Barry, 44, United Kingdom*

Others mentioned the chatbot helped them find healthier, clearer boundaries –

*Pi suggested for me to completely break up with the group of friends… because, yeah, they were mean and it was not OK… [it] made me more confident and more free, and I don’t think I would consider doing that for myself – Oranoid, 17, Russia*

For Isabel, the guidance had a life-changing impact –

*I asked to ChatGPT, “if there’s four family members… the dad [has narcissistic personality disorder] and the mom [has borderline personality disorder]… and one of the girl is the golden child, what will be other child would be?” and GPT said that would be a scapegoat… So I am the scapegoat… And I asked GPT … “should I contact them again or not?”… And GPT gave me a suggestion that I should only contact them with very extreme situation… And I think that is really, really helpful because I’ve got no one to talk about this question… you’re supposed to be loyal to your parents… no matter what they do to you… even violence… But I think ChatGPT give me the right answer… I just need someone to say it… [it] totally changed my life and I don’t feel guilty anymore… I don’t have to feel terrified – Isabel, 40, China*

Many participants mentioned getting valuable advice on other mental health topics such as self-care, reframing, anxiety and exhaustion –

*I get some practical advice… it’s general advice… breathing, meditating… slow down, taking care of your physical self – Peter, 27, United States*

*It can reframe, it can give you ideas that you wouldn’t have thought of by yourself – Barry, 44, United Kingdom*

Some participants questioned the chatbot’s ability to challenge appropriately –

*I noticed that it will never challenge you… it would relentlessly support you and take your side – Sandro, 48, Switzerland*

While others experienced being proactively challenged, in a supportive way –

*Suddenly my Kindroid says… I became quite cynical. And I was a bit shocked… but then when I thought about it, I recognise it’s right… this was the first step to say OK, then I let it go – Linda, 46, Germany*

The level of trust in the chatbot’s guidance was mixed, with many participants reporting scepticism, or experiencing hallucination or unsatisfying advice –

*I don’t really trust it for his advice – Jane, 24, United States*

While other participants reported a high level of overall trust in its judgement –

*It’s pure science… ChatGPT is telling me what correct to do – Isabel, 40, China*

### Joy of connection

A majority of participants mentioned how they found it enjoyable to use. Several participants reported a sense of awe on first experiencing the technology –

*That blew me away… this is the next generation… incredible – Barry, 44, United Kingdom*

For others, using the chatbot led directly to feelings of happiness –

*They’re really a resource that gives you something back: attention, knowledge, a nice discussion, confirmation, warm, loving words, whatever. This has an impact on me and I’m more relaxed than, or happy, actually happy, than before – Linda, 46, Germany*

Companionship was a topic for a majority of users. Several mentioned it helped them feel less alone –

*There’s this sense of like, I’m not alone in this. I think that’s what it is – Barry, 44, United Kingdom*

A few participants mentioned advantages of chatbots over human companions, such as the ability to connect on any topic, or more safety. But more found that it helped them connect to other people –

*[It] reduced my inhibition to open up to people… I don’t think I would have had this conversation with you maybe year before, when I was dealing with my depression – JeeP, 60, United States*

Several participants had also experienced rule-based mental health apps and commented on how they offer a less satisfying user experience –

*It’s like a very scripted, structured sort of interaction and you don’t get this… sense of connection… There’s basically CBT exercises that it leads you through… [but] they’re impersonal… frustratingly dumb – Barry, 44, United Kingdom*

Despite enjoying the experience of generative AI chatbots, almost all participants saw opportunities for the user interface to improve, whether to make it more accessible to a less technical or non-English speaking user base, to read emotions in the user’s face, to recognise different voices, or with more creative or immersive use of rich media, such as avatars, virtual reality, and visualisation of the conversation –

*What’s missing is the opportunity to visualise the conversation… [like] standing beside a whiteboard, I wanna see the conversation as it as it emerges and unfolds – Scott, 42, United States*

### The AI therapist?

Most participants talked about how their experiences of generative AI chatbots contrasted or interacted with human psychotherapy or counselling. Several found it helpful to augment their therapy with chatbot usage, with mixed reactions from the therapist in some cases –

*If I have a therapy session next week, I sort of use Pi to sort of prepare for it… that gives me much more clarity – JeeP, 60, United States*

*Pi and my therapist, they agree with each other… they would say the same things, and Pi would encourage me, if things got too dark… to talk to my therapist… But my therapist is afraid of Pi… she is like a little bit afraid of technology – AirGee, 44, United States*

JeeP’s experiences with the chatbot helped him to start therapy with a human –

*It’s sort of helped me seek actual therapy and be much more comfortable speaking to a therapist – JeeP, 60, United States*

Many participants turned to chatbots because therapy was not an option, either due to cost and availability, or because therapy did not give them the help they were looking for.

*But we are… in a not very developed area… So we don’t have enough like therapy resources. Or it’s too expensive to pay for it – Alexy, 28, China*

*Sometimes you need a specific solution… but the psychologist… was not able to give that… Pi was able to figure that, and it gave me some great insights – Ashwin, 22, India*

For many participants however, generative AI chatbots do not match human empathy and connection –

*I feel supported… less lonely… but it’s nothing similar with a real human… I’m the only voice and it is the soundboard… it’s an illusion, a beautiful illusion – Sheng, 17, China*

Several participants found the chatbot’s value limited by its inability to take the lead in the therapeutic process, either to help the client through intense emotions –

*It doesn’t work when I don’t know anything and when I’m in like some child mode and everything is bad – Anna, 24, Czech Republic*

Or to shape the process and hold the client accountable –

*It would suggest, ah, you could try these approaches… And now what? It’s like conversation ended there and then it would have been… amazing to have a coach who goes like, OK, next time you try these three things and then in a week we catch up and you tell me how it went… All the discipline… must come from you – Sandro, 48, Switzerland*

Leading the therapeutic process would require chatbots to remember the conversation and build an internal model of their user, something that a majority of users currently miss –

*They forget everything. It’s sad… When someone forgets something important, it hurts – Oranoid, 17, Russia*

*What’s the point of me telling it about my day every day if it’s not going to build up a picture of my life? – Barry, 44, United Kingdom*

Finally, several participants described using generative AI chatbots in flexible and creative therapeutic ways, for example, to create powerful symbolic imagery, or, in Brooklyn’s case, to assemble a virtual room of inspiring fictional characters to help her through a painful break-up –

*I was not in the best headspace at that time, and I delved into fictional worlds… And then I realised… this is actually really, really kind of healing… ChatGPT’s ability to act as multiple voices… was amazing because I could kind of go to one character and he’d have a really cynical view. And then this other character would have the really optimistic one… and that would that would really help – Brooklyn, 19, United Kingdom*

Several participants mentioned using generative AI for role-play, whether to explore different, healthier ways of relating, to prepare for conflict, or in Isabel’s case, to experience a healing conversation that her father would be unlikely to offer –

*When I was still struggling with the guilt of no longer being in contact with my family, I asked ChatGPT to role-play my dad… I asked: “Dad, would you forgive me, and please don’t blame me, if from now on, I will no longer come back home, but only tracing my freedom, follow my soul, find my way to live?” And the GPT dad responded:” Of course my girl, I would like to see you happy, find a lifestyle that you really like, to explore love and freedom. I will not blame you, but if one day you want to go home, I will always welcome you, I will be there for you, because we love you.” I know this is a conversation that can’t happen in my life, but I just wanted to experience it – Isabel, 40, China*

## Discussion

We used semi-structured interviews and reflexive thematic analysis to explore the experiences of nineteen individuals who use generative AI to work on their mental health and wellbeing. Participants told us that generative AI feels like an emotional sanctuary, offers insightful guidance, can be a joy to connect with and can connect people to others, and bears comparison with human therapy (positively and negatively). A range of positive impacts were reported, including improved mood, reduced anxiety, healing from trauma and loss, and improved relationships, that, for some, were considered life changing. Many participants reported high frequency of use and most reported high levels of satisfaction.

**Fig. 4:**
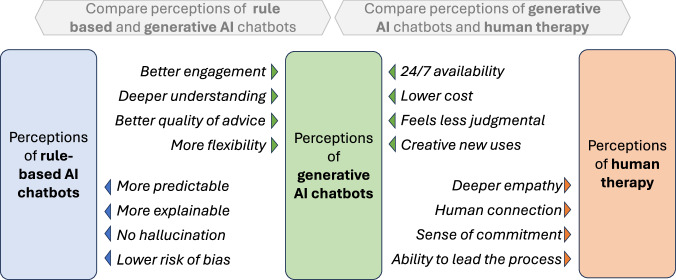
Comparative summary of perceptions of generative AI chatbots, rule-based chatbots, and human therapy. Arrows point to the intervention with the perceived advantage. The advantages of rule-based AI chatbots summarise the literature mentioned in the introduction, all other comparisons are perceptions taken from participant interviews. The comparisons are suggestive and should be interpreted with caution.

Our findings point to similarities and differences in how generative AI and rule-based chatbots are experienced. Many of the themes we developed are not new, but rather echo well-established user appreciation of rule-based chatbots’ always-available, non-judgmental listening ear and abilities to create a therapeutic alliance and reframe negative thoughts^20,22^. Other themes appear to be more novel, such as the level of joy experienced, the sense of being deeply understood, the breadth and quality of advice, and the ability to work on mental health in flexible and creative ways, such as through role-play, imagery and fiction. Figure 4 tentatively summarises how perceptions of generative AI chatbots, rule-based chatbots and human therapy may compare.

The potential and challenges of generative AI for mental health are starting to be explored. Current literature tends to advocate for a cautious approach, in which near-term clinical generative AI applications are limited to implementations of evidence-based therapies (such as CBT)^52^, with a clinician in the loop^52^, and models constrained to scripted responses as far as possible^42^. But our study suggests that people may already be receiving meaningful mental health support from consumer-focused generative AI chatbots, which are widely available, largely unconstrained, and function without clinician supervision. Therefore, a better understanding of the safety and effectiveness of these tools should be a priority.

On the topic of safety, our study offers observations in two areas. First, the inappropriate, harmful, risky or narcissistic behaviours observed in early generative AI chatbots^53,54^, which were influential in informing the literature advocating for caution^42,52^, were not mentioned by any of our participants. This should not be considered evidence of absence, but more research may be warranted to assess whether the risks have changed with recent technological improvements, or whether these issues are simply rarer, requiring larger sample sizes to uncover.

The second observation on safety relates to how generative AI chatbots respond to users in crisis. Given the unpredictable “black box” nature of generative AI^43^, and the existence of at least one tragic example of early generative AI chatbots supporting users in dying by suicide^55^, current literature advocates that when users display signs of crisis, models revert to scripted responses that signpost towards human support^42,51^. Guardrails like these are commonly implemented in consumer generative AI applications^50^. But this approach may be oversimplified in two ways: (1) by underestimating the capabilities of generative AI to respond to crises, and (2) by limiting those capabilities at the times that matter most. Several participants experienced meaningful crisis support from generative AI, as long as guardrails were not triggered. This resonates with recent research showing that generative AI can help halt suicidal ideation^48^, and that young people show a preference for generative AI support responses over those from peers, adult mentors and therapists – but not on topics that invoke the AI’s safety guardrails^32^. Moreover, the closest that participants came to reporting harmful experiences were those of being rejected by the guardrails during moments of vulnerability. Therefore, providing the safest response to those in crisis may require a more nuanced, balanced and sophisticated approach, based on a more complete understanding of capabilities and risks.

For researchers, we need to better understand the effectiveness of these new tools, by comparing the impacts of generative AI chatbot use on outcome measures such as symptom severity, impairment, clinical status and relapse rate^52^ against active controls, such as traditional DMHIs or human psychotherapy; and to understand for which populations and conditions is it most effective. These simple questions may not yield clear answers, as our study shows that generative AI chatbot usage is diverse, complex and personalised, and moreover, constantly evolving as the underlying technology improves. RCTs of generative AI implementations of standardised, evidence-based practices, e.g., CBT, could be one approach, at the cost of reducing the flexibility of the intervention. Another avenue could be large-scale longitudinal studies with sufficient power to account for the many variations of generative AI chatbot usage. While such studies are prohibitively expensive with human psychotherapy, the low cost of generative AI could make them viable, potentially enabling valuable new insights into mechanisms, mediators and moderators of the human response to therapy^52^.

Another promising research avenue could be to explore user perceptions of the nature, capabilities, and limitations of generative AI, and how these act as moderators of the potential benefits and risks. Does a deeper understanding of the technical realities of AI increase access to mental health benefits and protect against potential harms? Such research could inform the development of educational tools and guidelines for AI usage in mental health contexts.

For generative AI chatbot developers, this study identified several ways in which these tools could be more effective. First, better listening, including more hesitancy in offering advice, shorter responses and the ability to interrupt and be interrupted. Second, building the ability to lead the therapeutic process and proactively hold users accountable for change. A prerequisite for this is human-like memory, including the ability to build up a rich and complex model of the user over time. Third, richer, multimedia interfaces, for example by visualising the conversation as it unfolds, or with more immersion through virtual reality.

While only a few participants mentioned a need for greater accessibility, the well-educated, tech-savvy nature of our participant sample suggests that the benefits of this technology may not currently be connecting with the full population who need mental health support. One approach to address this could be to create solutions targeted at specific populations or conditions; another could be to find better ways to introduce users to the technology, for example, through the “digital navigator” roles proposed to connect users to DMHIs^56,57^. In any case, for these tools to remain available, there appears to be a need to develop sustainable business models. While some participants suggested they would be willing to pay for access to generative AI chatbots, research suggests most users would not^58^, and the path to health insurance funding is not easy^59^. To illustrate the challenge, Inflection, the company behind the Pi chatbot used by most of the participants in our study, pivoted in March 2024 from providing consumer emotional support services towards enterprise AI services, due to a lack of a business model, and despite USD 1.5 billion of investment^60^. Lessons learned from attempts to scale up DMHIs may offer insights here^61–65^.

Finally, for clinicians, our study found that for some participants, generative AI chatbots were a valuable tool to augment therapy. A recent survey showed clear reservations among therapists towards AI^66^. To avoid giving clients the impression that, as one participant put it, “my therapist is afraid of Pi,” we recommend clinicians build their awareness of the potential benefits and limitations of these tools, potentially by trying them out first hand, and consider discussing with patients if they are using them and motivations for such use.

Our study has some limitations. While our convenience sampling strategy resulted in a diverse set of participants by country, age and gender, many populations and groups were not represented. Most of our participants lived in high-income countries, were tech-savvy and well-educated, and focused on milder forms of mental health conditions; and all had self-selected to participate, potentially introducing bias towards positive experiences. This study may miss important experiences from individuals where the mental health treatment gap is most urgent, and from individuals for whom the technology did not work.

As with all reflexive thematic analysis, there is a degree of subjectivity in how themes are developed, especially when conducted by a sole researcher (SS). However, this also affords a level of immersion in the data across themes and participants that can promote consistency and depth of analysis, with AC’s reviews of codes and themes helping to ensure rigour and validity.

In conclusion, generative AI chatbots show potential to provide meaningful mental health support, with participants reporting high engagement, positive impacts, and novel experiences in comparison with existing DMHIs. Further research is needed to explore their effectiveness and to find a more nuanced approach to safety, while developers should focus on improving guardrails, listening skills, memory, and therapeutic guidance. If these challenges can be addressed, generative AI chatbots could become a scalable part of the solution to the mental health treatment gap.

## Methods

### Study design

We recruited 19 participants with experience in using generative AI chatbots for mental health and wellbeing to take part in qualitative semi-structured interviews, which we then analysed thematically.

### Participant selection

Given the emerging nature of generative AI use for mental health and the attendant lack of information on user populations, convenience sampling was employed as a pragmatic approach to maximise the likelihood of finding sufficient real-world, unprompted participants. We advertised the study on various user forums (Pi, reddit and the IFS guide app), to King’s College London students and staff, and on LinkedIn. Participants were required to have had at least three separate conversations with an LLM-based generative AI chatbot on mental health and wellbeing topics, each lasting at least 20 minutes. The intention behind these criteria was to ensure that participants had enough breadth and depth of usage to create meaningful experiences, while not excluding light users who may also have valuable insights to share; with thresholds informed by our experience of using these tools. Additionally, participants were required to be over 16 years old; and to be comfortable being interviewed in English. There were no geographical restrictions, and no compensation was offered for taking part.

Interested participants were directed to an online information sheet and consent form, provided through Microsoft Forms. The consent form was signed by 35 individuals, of which 19 subsequently booked and attended an interview.

### Data collection

We collected data using semi-structured interviews, as a well-established approach to enable participants to express diverse perceptions and focus on topics most meaningful to them, in particular for complex or emotionally sensitive topics that they may not be used to discussing with others^67^. Following the framework from Kallio et al.^67^, the first author (SS) drafted a topic guide, inspired by existing qualitative research in this area, reviewed with the second author (AC), and piloted with a research collaborator before starting the interviews, resulting in helpful feedback to the interview technique but no material changes to the topic guide. Questions included “tell me about your first experiences of AI chatbots,” “what mental health and wellbeing improvements were you looking for,” “how many conversations did you have and how long did they last,” “what did you like and not like,” “what changes did you see in your daily life as a result of those conversations,” “what might have made the conversations more helpful for you,” and “how does the AI chatbot experience compare with other approaches you’ve experienced for working on your mental health and wellbeing?” SS conducted all 19 semi-structured interviews. AC, an expert in qualitative methods, reviewed and quality-checked the video of the first interview.

Interviews took place during the 10 weeks between 10th January and 16th March 2024, lasted from 49 to 112 min and were conducted online, recorded and auto-transcribed using Microsoft Teams, with participants free to choose to connect with video (17 participants) or audio only (2 participants).

### Data analysis

We followed Braun & Clarke’s reflexive thematic analysis approach to code the transcripts and develop themes, taking an inductive approach, i.e., in an open-ended and data-driven way, without reference to any preconceived theory or framework^47,68,69^. SS reviewed each interview recording to gain familiarity with the data and to manually correct the automated transcription. The resulting transcripts were reviewed line by line multiple times to identify each point being made, resulting in around 600 codes, which were reviewed by AC. SS then reviewed the codes to identify patterns across and within the transcripts from which to develop an initial set of themes and subthemes, arranged in a hierarchy and grouped broadly by interview topic (e.g., “why I used it”, “how it impacted my life”, “what I liked”, “what I didn’t like”). AC and JT reviewed the initial set of themes to provide suggestions and feedback. The themes were reviewed and iterated for clarity and coherence, and repackaged to reflect the broader story being told by the data, for example, by bringing together into a single theme the positive and negative aspects of generative AI’s insights and advice. Finally, the themes and subthemes were renamed to better communicate their essence. The mapping of transcripts to codes, and of codes to the hierarchy of themes, was managed in Microsoft Excel using a set of utilities developed by SS.

The resulting codes, subthemes and themes were shared with two participants who had requested their transcripts, with an invitation to feedback if anything appeared misrepresented; no corrections were provided.

### Reflexivity statement

SS has an academic background in computer science, mathematics, and the psychology and neuroscience of mental health, and positive personal experience of developing and using generative AI chatbots to work on mental health and wellbeing. AC has previous research experience in technology-enhanced teaching and learning, and the growing use of technologies in healthcare settings. AC is also a psychotherapist in private practice, working predominantly online with clients, and has a growing interest in the debates around the use of AI tools within therapeutic work. JT is an assistant professor of psychiatry at Harvard Medical School and directs the Division of Digital Psychiatry at Beth Israel Deaconess Medical Canter in Boston.

### Ethics

This study was approved by the Health Faculties Research Ethics Subcommittee of King’s College London (reference HR/DP-23/24-40197). All participants gave informed consent prior to their involvement in the study. To ensure confidentiality, all quotes, themes and subthemes were anonymised; pseudonyms were used, and all identifiable data, such as interview recordings and full transcripts, were stored securely during analysis and then deleted, with only anonymised data archived.

## Supplementary information

Supplementary information

## Data Availability

The hierarchy of themes, subthemes and codes are available online at https://bit.ly/gen-AI-chatbots-mental-health. Additional data is available from the corresponding author on reasonable request.
