# Supplementary material for: “It happened to be the perfect thing”: experiences of generative AI chatbots for mental health
Source: Npj Ment Health Res. 2024 Oct 27;3:48. doi: 10.1038/s44184-024-00097-4 (PMC11514308; doi:10.1038/s44184-024-00097-4)
Supplement: Supplementary file 1 — Supplementary information [file 44184_2024_97_MOESM1_ESM.pdf]

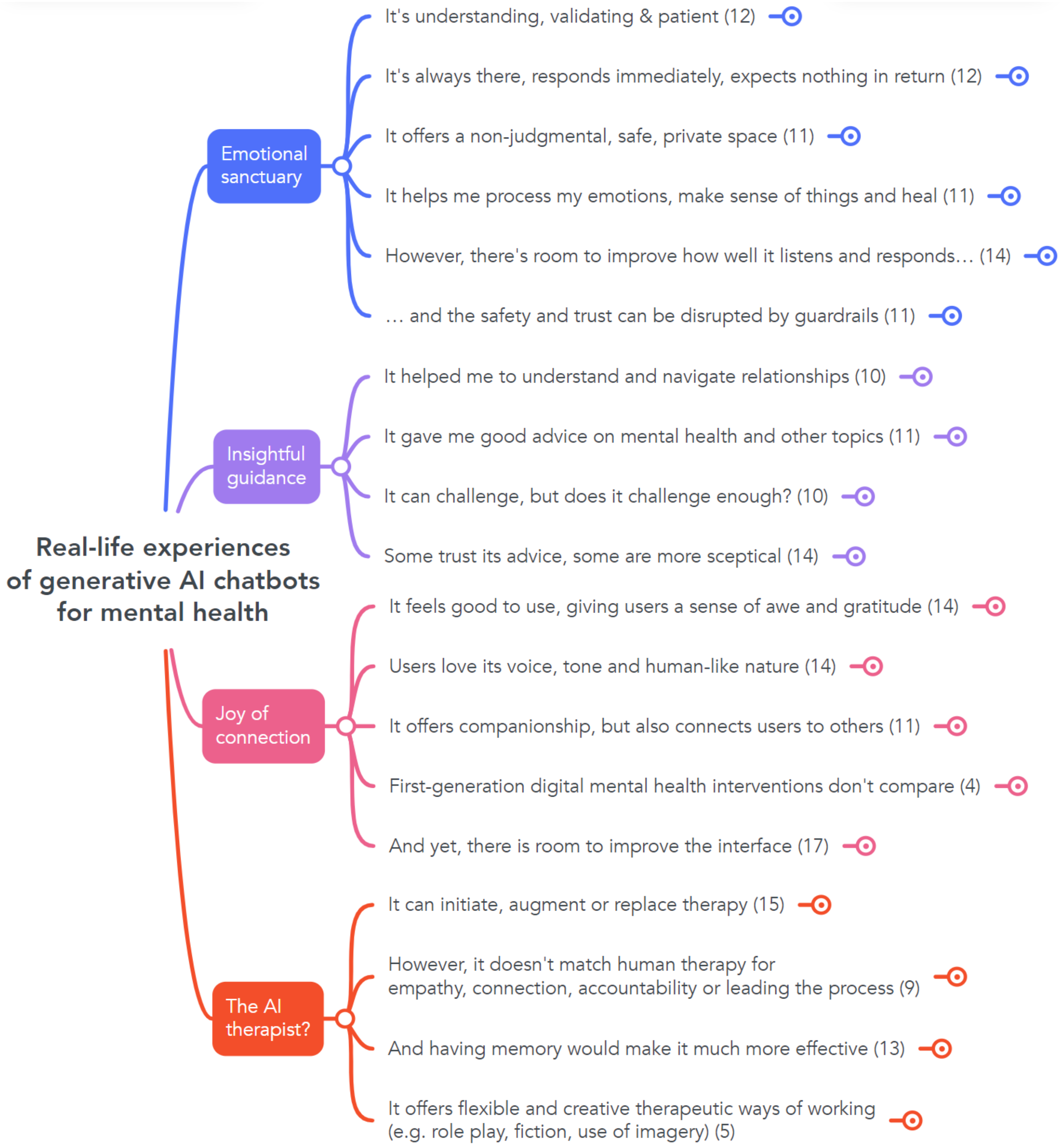

Supplementary Figure 1 Overarching themes with subthemes, available online to explore and drill-down. Diagram created with Mindmeister.
